# Roll Angle Estimation of a Motorcycle through Inertial Measurements

**DOI:** 10.3390/s21196626

**Published:** 2021-10-05

**Authors:** Diego Maceira, Alberto Luaces, Urbano Lugrís, Miguel Á. Naya, Emilio Sanjurjo

**Affiliations:** Laboratorio de Ingeniería Mecánica, University of A Coruña, 15403 A Coruña, Spain; diego.maceira@udc.es (D.M.); alberto.luaces@udc.es (A.L.); urbano.lugris@udc.es (U.L.); miguel.naya@udc.es (M.Á.N.)

**Keywords:** roll angle estimator, Kalman filter, LQR controller, inertial sensors, motorcycle lean angle

## Abstract

Currently, the interest in creating autonomous driving vehicles and progressively more sophisticated active safety systems is growing enormously, being a prevailing importance factor for the end user when choosing between either one or another commercial vehicle model. While four-wheelers are ahead in the adoption of these systems, the development for two-wheelers is beginning to gain importance within the sector. This makes sense, since the vulnerability for the driver is much higher in these vehicles compared to traditional four-wheelers. The particular dynamics and stability that govern the behavior of single-track vehicles (STVs) make the task of designing active control systems, such as Anti-lock Braking System (ABS) systems or active or semi-active suspension systems, particularly challenging. The roll angle can achieve high values, which greatly affects the general behavior of the vehicle. Therefore, it is a magnitude of the utmost importance; however, its accurate measurement or estimation is far from trivial. This work is based on a previous paper, in which a roll angle estimator based on the Kalman filter was presented and tested on an instrumented bicycle. In this work, a further refinement of the method is proposed, and it is tested in more challenging situations using the multibody model of a motorcycle. Moreover, an extension of the method is also presented to improve the way noise is modeled within this Kalman filter.

## 1. Introduction

Single track vehicles (STVs) present some intrinsic advantages as a mobility solution: they are lighter, use less space, and have better fuel economy, particularly at low speeds. These advantages make them great candidates for urban mobility. However, due to their lack of static stability, they also present some intrinsic challenges regarding safety and autonomous driving.

One magnitude that is of particular interest is the roll angle with respect to gravity (i.e., with respect to the vertical), since it is the magnitude used to keep the balance of the vehicle when it is in motion. Therefore, any control system that aims at controlling the balance of a STV, or any advanced driving aid for human-driven vehicles, has to take the roll angle into account. The roll angle determination has other potential applications.

For example, it is required in scientific experiments, such as those performed for the rider control parameter identification [1] or to evaluate the maneuverability of a motorcycle [2]. Unfortunately, there is not any means of measuring the roll angle of a STV in an economic and reliable way. Usually, the practical approach is measuring this magnitude indirectly through state observers, which combines the information provided by some sensors with a mathematical model to estimate the variable of interest. Consequently, this problem has been studied for several years, trying to improve the estimation algorithms.

There are some works using video-based approaches or distance sensors. The roll angle measurements using these types of devices are not reliable enough to be installed on commercial vehicles, since they are sensitive to the dirt. Moreover, they provide roll angle estimations with reference to the ground (video-based approaches use the horizon line (e.g., [3]), and distance sensors use the distance to the ground).

However, in order to keep the balance of a STV, the roll angle with respect to gravity is needed. Therefore, we will focus on works relying on inertial and odometric sensors, since they are already widespread in the automotive field for safety applications. The reason is that they are affordable and reliable because they do not depend on the lighting (as do the video-based approaches), the ground properties, and geometry (as do the distance sensors), nor the sky visibility (as does Global Positioning System (GPS)).

In [4], an extended Kalman filter (EKF) based on an analytical dynamics model was presented. This model is quite complex, and hence it can provide a lot of information of the vehicle; however, at the same time, the estimations rely on a correct characterization of the multiple parameters of the model. Moreover, the parameter values have to be adapted if applied to a different model of motorcycle. Similarly, in [5], a dynamics model of a motorcycle is used into an observer, although the latter is an unknown-input high-order sliding-mode observer instead of an EKF. The fuzzy logic approach has also been applied to the estimation of the states of motorcycles using dynamical models [6].

However, whenever possible, simpler models are preferred, since they are easier to adjust, and serve a broader range of vehicles with only minor or no tuning at all. The following works belong to this category. In [7,8], an observer based on the frequency separation is presented and validated. In [9], an EKF and an unscented Kalman filter (UKF) were compared, achieving a similar level of accuracy. In [10], another EKF based on inertial measurement was presented. It was applied to a racing motorcycle and validated on racing tracks. All these methods, and some other similar works not cited here, are compared with different tracks, sensors, vehicles, and riding conditions. Therefore, it is difficult to make an objective comparison of their effectiveness.

The present work is based on the roll angle estimation method presented in [11], where a roll angle estimator based on the EKF and angular rate measurements was validated at low speed on a flat floor with a bicycle. Due to limitations of the experimental setup, only low speeds were considered in leveled roads, with thin tires and no suspensions and without knowledge of the rider motions. In order to fill the gaps left by [11], in this work, a multibody model of a motorcycle is developed.

This multibody model is covered in Section 2, including the force models, such as toroidal wheels, suspensions, and a rider that can move laterally to displace its torso inwards or outwards during the turns, which are covered in Section 2.2. In order to complete the desired maneuvers, some controllers are needed, where the drive and braking forces are controlled by longitudinal controller, and the steer torque is governed by a lateral controller, which is in charge of keeping the motorcycle balance while following the predefined trajectories. These controllers are covered in Section 2.3.

Then, Section 2.4 describes the sensor models obtained from the multibody simulations. A sequence of pseudorandom noise is added to the signal of every sensor to provide realistic noise properties. The multibody model is run in several scenarios, which are described in Section 2.5. Finally, two roll angle observers based on the one presented in [11] are developed and tested in Section 3 using the noisy signals previously obtained from the multibody simulations. The roll angle estimations are compared in Section 4 to the roll angle obtained by the multibody simulation, which is used as the reference in this work.

## 2. Methods

This work is focused on the estimation of the roll angle of a motorcycle. In order to achieve this, a multibody motorcycle model is developed. Sensor models are implemented on the multibody model, and these data are used to run a state observer that estimates the roll angle of the multibody model. Since the sensors of the multibody model are “perfect”, a sequence of pseudorandom numbers is added to mimic the noise of a realistic signal.

This section deals with the description of the multibody model, including its kinematics and the main force models, namely, tire forces, suspensions, drive and brake forces, etc. Since the motorcycle is an intrinsically unstable system, a controller is required to maintain the balance and follow the prescribed trajectory, which is also described in this section. There are two more controllers: a longitudinal controller, which adapts the speed of the motorcycle depending on the curvature of the upcoming path, and a rider controller, which is used to control the position of the rider’s torso, which can be in a neutral position, tilting inside the curve, or tilting outside the curve. Finally, this section describes the sensor data obtained from the multibody model and the designed tracks where it is simulated.

### 2.1. Multibody Model

**Figure 1:**
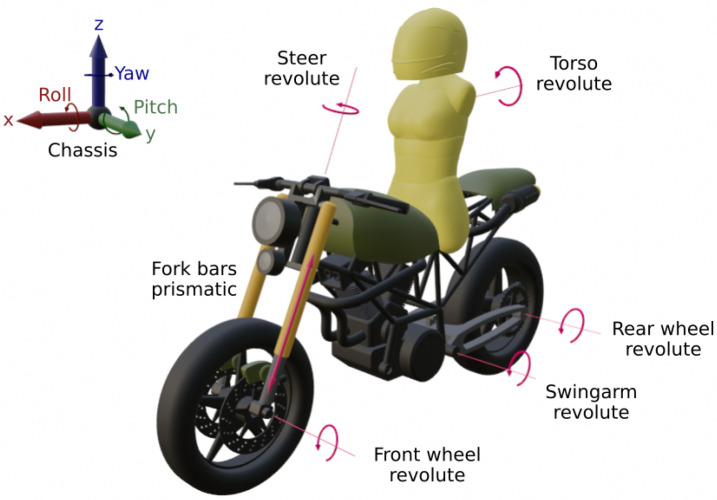
Multibody model elements and DOFs.

The model used is a seven-element assembly without closed kinematic loops, where six elements belong to motorcycle parts, and one of them represents the driver’s torso. As a result, a 12 Degrees of Freedom (DOF) model is obtained: six DOF from the chassis rigid body condition, five revolute joints from the two wheels, swingarm, steer and torso roll movement, and one prismatic joint between the fork bars and the fork bottles. All the motorcycle elements and DOFs are represented in Figure 1.

Mass and inertia properties for each element of the model are shown in Table 1. Some of them are taken from the bibliography [12] and other are obtained from a CAD tool.

A relative coordinates formulation was used, which is a well suited option to take advantage of the topology of the mechanism. Each solid in the chain is defined relative to the previous one, using algorithms to calculate kinematics and dynamics terms by means of recursive methods. An in-depth description of this formulations is covered in [13]. The trapezoidal rule was chosen as the integrator, with a 1 millisecond fixed time step to solve the dynamics.

### 2.2. Force Models

Some forces are taken into account to achieve a realistic behavior of the motorcycle and its dynamics, such as gravity influence, forces from the motorcycle subsystems, such as brakes, tires and suspensions, and different torques applied to rear wheel, to steer mechanism and to the driver’s torso. Rear wheel torque represents power transmission as an in-wheel hub motor would produce, steer torque represents the rider input to control the vehicle, and torso torque aims to represent the influence of the rider’s movements in the roll angle estimation.

#### 2.2.1. Brake Models

The brake model employed in this research is based on the tangential force model developed in [14], which takes into account sliding and stiction phenomena, with original contributions from [15]. In essence, a dry friction model is used in series with a spring-damper model. When the spring force exceeds the maximum achievable braking torque for a given braking pressure, one of the ends of the spring is allowed to slide so that the maximum braking force is not exceeded.

The front wheel has more braking capabilities than the rear one, as in a conventional motorcycle, but both brakes act together when braking. The maximum brake torque applied to each wheel is obtained by estimating the longitudinal force that the tires can perform according to Equation (1).

(1) $$F_{long}=\mu N,\;$$

where μ is the tire-friction road considered, and *N* is the vertical force supported by the wheel. In case of the front wheel, this force is considered to be the whole weight of the vehicle and the driver, since in an emergency braking situation, almost all the weight rests on the front wheel. The normal force considered for the rear wheel is the weight that it supports when going straight and without any longitudinal acceleration.

#### 2.2.2. Tire Models

Tire behavior and properties play a crucial role in the evaluation of the motorcycle dynamics. In this work, toroidal tires are used, since it is a good approximation to the behavior of motorcycle tires. They are defined with an outer radius, *R*, which represents the undeformed tire outer radius, and the torus tube radius, *r*, which should be selected to represent, in the most accurate way, the tire curvature near the contact patch.

Tire force models are divided between the normal force model and tangential force model. Normal forces calculation is closely related with the contact routine used to detect the intersection between the tire and the floor. In this research, a triangle mesh was used to characterize the floor, and thus the first step to calculate normal forces is to solve the contact problem between the torus and the triangle mesh. The contact algorithm between analytic torus and the triangle mesh used in this work is described in [16]. After a wheel contact is detected, the normal force is modeled as a spring-damper force. However, the force of the spring-damper element is limited such that it can produce compression forces over the road, but traction forces are not allowed.

Related to the tangential force model, part of the TMeasy tire model was applied [17], using the same simplifications as in [18]. This is an empirical and physical tire model, in the sense that first, curve fitting using few parameters is necessary to adjust the tire characteristic curves, and then dynamic behaviors of the tire are considered. The model takes into account both the longitudinal and lateral forces experienced by the tires, and these forces are slip-dependent in both directions.

**Figure 2:**
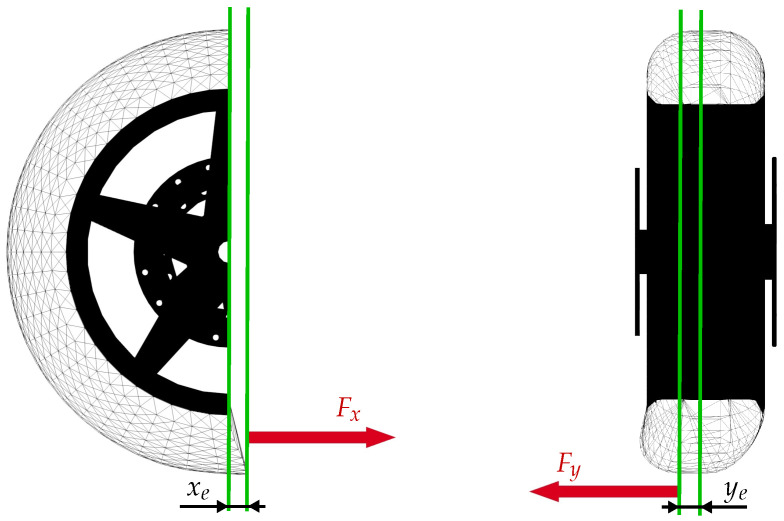
Longitudinal an lateral deflections in a toroidal tire due to tangential forces.

As a consequence, the effects of tire deflection affect the tire behavior when the forces vary. This phenomenon is specially important when the vehicle moves at very low speed, because small displacements can produce high slips. If tire deflection is not considered, these high slips introduce unrealistic high forces. What actually happens is that the forces acting in the contact patch deflect longitudinally and laterally the tire as shown in Figure 2.

#### 2.2.3. Suspension Models

The motorcycle has traditional suspension systems for this kind of vehicles. Thus, for the front assembly, an inverted telescopic fork was chosen. For the rear frame, a monoshock absorber links the swingarm and the chassis. In both cases, a linear spring-damper model is used.

#### 2.2.4. Drive Torque

The model simulates power transmission from an electric motor by introducing a torque in the rear wheel. Torque values have a dependency with angular rate, hence tabulated values with realistic properties are used. This torque value will be calculated by the longitudinal motorcycle controller, which will be described in depth in Section 2.3.2.

#### 2.2.5. Steer Torque

Steer torque is the only input used to control the lateral dynamics of the motorcycle. It is used to control both the lateral equilibrium and to follow the prescribed trajectory. It highly affects the forces experienced by the tires, modifying completely the whole vehicle dynamics. This variable is managed by the lateral motorcycle controller, which will be covered in Section 2.3.3.

#### 2.2.6. Torso Torque

This work aims at analyzing the rider’s influence on the estimation of the vehicle roll angle. For this purpose, a torque between the torso and chassis elements is introduced in order to verify if the estimation is influenced by these movements. There is a specific controller managing this torque value, and it will be explained in Section 2.3.4. In this work, the torso controller is not used as a tool for motorcycle stabilization.

### 2.3. Controller Models

Since the aim of this work is to test the roll angle estimation in different conditions and scenarios, the motorcycle model has to be able to perform in a wide variety of circumstances. To achieve that, several controllers were implemented. First, longitudinal control manages accelerating and braking phases. Secondly, lateral control guarantees the lateral dynamic equilibrium while following the specified trajectories. The longitudinal controller has to be able to keep the velocity, which allows the lateral controller to perform the desired maneuver.

Lastly, a torso controller is used to perform maneuvers with a lean relative angle between torso and motorcycle, inside and outside the turns, as an option.

#### 2.3.1. Path Tracking

In order to follow a predetermined path, it is necessary to use a curve definition system that can encode the trajectory that the motorcycle has to follow. In this work, splines are employed. Each scenario has a finite number of splines. They are drawn using the Blender software (Blender Foundation, Amsterdam, The Netherlands), and then their parameters are exported to a text file to be read by the simulation program.

Then, a point referred to the chassis frame is defined, and its position is compared to the path previously defined. This point is not necessarily coincident with the chassis. In this work, it is ahead of the chassis position to evaluate the deviation with respect to the trajectory in advance, as a human driver would do. This reference point position is speed-dependent: the faster the motorcycle goes, the farther and higher the point is found, as shown in Equations (2) and (3), where $v_{ch,x}$ and $v_{ch,y}$ stand for chassis velocity components, and $rp_{x}$ and $rp_{z}$ are the coordinates of the reference point expressed in the local reference system of the chassis (in SI units). In this work, the position of this reference point has an important effect on the overall behavior of the controller.

(2) $$\begin{array}{ccc} rp_{x} & = & 0.75\sqrt{v_{ch,x}^{2}+v_{ch,y}^{2}} \end{array}$$

(3) $$\begin{array}{ccc} rp_{z} & = & 0.1\;rp_{x} \end{array}$$

The constants were adjusted by trial and error. The height of the reference point is also variable with speed. This height is relevant in tilting vehicles, since it produces a lateral displacement of the reference point when the vehicle is tilted. Once this point is defined, it is necessary to calculate the distance between the point and the nearest spline of the track. In this work, we calculate the distance to the spline perpendicular to the longitudinal axis of the motorcycle model.

With this information, the position, velocity, and orientation errors can be calculated, in order to correct the motorcycle position with respect to the desired path. These error values are the data that the lateral controller needs to calculate the roll angle target required to correct the motorcycle trajectory respect to the desired path. This will be addressed in Section 2.3.3.

#### 2.3.2. Longitudinal Controller

This controller manages the acceleration and braking phases. The longitudinal control is designed to perform the maneuver as fast as possible, taking into account the curvature of the trajectory and the power limits of the motorcycle. The controller evaluates the path ahead the motorcycle 10 s in advance with respect to the reference point described on the previous section, and thus there is enough time to start braking before arriving too fast at a turn. This value is translated into a distance variable by means the Equation (4), which means the position in advance is calculated taking into account the current speed of the motorcycle.

(4) $$x_{i+t_{preview}}=t_{preview}\;v_{i}$$

Once the position in the spline is known, the maximum speed is obtained in relation to the curvature of the path (κ), by means of the steady-state cornering equilibrium equation [19], expressed in Equation (5), where $\phi_{max}$ stands for the maximum roll angle the motorcycle can achieve, which is a configuration parameter for the controller.

(5) $$V_{max}=\sqrt{\frac{g\tan\phi_{max}}{\kappa }}$$

The value of $V_{max}$ cannot achieve infinite values and is limited to a max value, which is a configuration parameter. Once known, the current speed is evaluated against it, and the controller will accelerate if the current speed value is lower than $V_{max}$, or will brake if the motorcycle speed is too high. Both of the actions are managed by proportional controllers, as shown in Equations (6) and (7).

(6) $$\begin{array}{ccc} accel & = & K_{acel}\;\left(V_{max}-V_{i}\right) \end{array}$$

(7) $$\begin{array}{ccc} brak & = & K_{br}\;\left(V_{i}-V_{max}\right) \end{array}$$

When accelerating, the value of accel is translated into a rear wheel torque, taking into account tabulated torque values and the wheel angular rate. When braking, the value of brak is transformed into brake torque in both wheels, but with different values, since the brake power capabilities are different, as mentioned before.

#### 2.3.3. Lateral Controller

**Figure 3:**
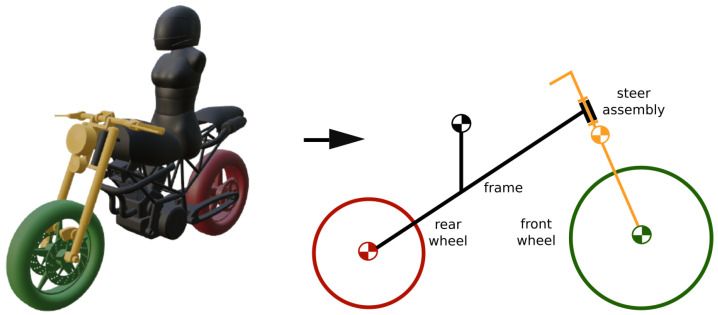
Multibody model transform to Whipple’s model.

In order to obtain full stability during maneuvers, a lateral controller was implemented. For this purpose, a Linear Quadratic Regulator (LQR) controller [20] was used, and a good behavior was achieved. Since the LQR controller requires a dynamic model in state-space form, the motorcycle multibody model was adapted into a Whipple model, and thus the initial seven-solid model is transformed into a four-solid model, as described in [21]. Model adaptation is shown in Figure 3. From the Whipple model, only the lateral dynamics are used.

Linearized dynamic equations of the model are expressed by Equation (8), where M, $C_{1}$, $K_{0}$ and $K_{2}$ are obtained from a set of 25 parameters of the motorcycle (see Table 2), $q={\left[\phi \;\;\delta \right]}^{⊤}$ is a vector that contains roll (ϕ) and steer (δ) angles, $f={\left[T_{\phi }\;\;T_{\delta }\right]}^{⊤}$ is a vector that contains roll and steer torques (the roll torque is considered to be null in this work), and *g* and *v* stand for gravity acceleration and forward velocity, respectively. The employed values are shown in Table 2.

(8) $$M\ddot{q}+vC_{1}\dot{q}+\left[gK_{0}+v^{2}K_{2}\right]\;q=f$$

Equation (8) can be expressed in state-space form as shown in Equations (9) and (10), where *u* is the input vector, x is the state vector and *y* is the system output.

(9) $$\begin{array}{c} \dot{x}=Ax+Bu \end{array}$$

(10) $$\begin{array}{c} y=\mathrm{Cx}+Du \end{array}$$

As Equation (8) is second order with respect to time and Equation (9) is first order, some changes are necessary. Taking $f={\left[0\;\;T_{\delta }\right]}^{⊤}$, $x={\left[q\;\;\dot{q}\right]}^{⊤}$ and its derivative as $\dot{x}={\left[\dot{q}\;\;\ddot{q}\right]}^{⊤}$, Equation (11) is obtained.

(11) $$\left[\begin{array}{c} \dot{q}\\ \ddot{q} \end{array}\right]=\left[\begin{array}{cc} 0_{2\times 2} & I_{2\times 2}\\ -M^{-1}\left[v^{2}\;K_{2}+g\;K_{0}\right] & -M^{-1}\;v\;C_{1} \end{array}\right]\left[\begin{array}{c} q\\ \dot{q} \end{array}\right]+{\left[\begin{array}{cccc} 0 & 0 & 0 & {\left(-M^{-1}\right)}_{2,2} \end{array}\right]}^{⊤}T_{\delta }$$

Once the parameters of Table 2 are calculated, M, $C_{1}$, $K_{0}$ and $K_{2}$ can be obtained. The following values are the ones employed on this work.

(12) $$M=\left[\begin{array}{cc} 81.6343 & 4.2211\\ 4.2211 & 1.0320 \end{array}\right]\;K_{0}=\left[\begin{array}{cc} -119.8071 & -8.7515\\ -8.7515 & -3.6985 \end{array}\right]$$

(13) $$K_{2}=\left[\begin{array}{cc} 0 & 84.9797\\ 0 & 6.6004 \end{array}\right]\;C_{1}=\left[\begin{array}{cc} 0 & 60.8120\\ -2.2266 & 5.8313 \end{array}\right]$$

To ensure system stability, state feedback is used, defining the system input as a negative feedback of the state, as in Equation (14), finding a K matrix that stabilizes the system.

(14) u=−Kx

Stabilization is achieved if the real parts of all eigenvalues of the system matrix are negative. Thus, Equation (9) can be combined with Equation (14), obtaining:

(15) $$\dot{x}=\mathrm{Ax}-\mathrm{BKx}=\left(A-\mathrm{BK}\right)x$$

Now, system stability is determined by the eigenvalues of A−BK, and thus a K matrix can be calculated with the values that ensure stability, since A and B are constants. In LQR controllers, the value of K is the one that minimizes the following cost function:

(16) $$J=\int_{0}^{\infty }\left(x^{T}Qx+Ru^{2}\right)dt$$

The function combines the quadratic values, integrated over time, of the magnitudes that should be minimized: the states and the control inputs. Each of them is weighted by a term, Q for the states and *R* for the input. The values of this terms can be adjusted in order to assign more weight to the control effort (increasing *R* value), or penalizing more the state errors by increasing the values of Q.

The trajectory of the motorcycle can be controlled through its roll angle ϕ, and thus we need to set it to a certain value at every time step. However, the LQR controller defined thus far is only a regulator, i.e., it drives the states to zero. Since the resulting system is of type 0 [20], we need to transform it into a type 1 system, in order to make it capable to track a reference value of the roll angle. This can be achieved by adding an integrator at the input. Therefore, the system size increases, as a new state is added, ξ, which is the integral of the tracking error, which means the integral of the difference between the controller target (*r*) and its output (ϕ). The input *u* changes its value to:

(17) $$u=-Kx+k_{I}\;\xi$$

and the value of ξ should be calculated during the runtime as shown in Equation (18), where dt is the simulation time step.

(18) $$\xi_{i}=\xi_{i-1}+dt\left(r-\phi \right)$$

Hence, the system turns into Equation (19):

(19) $${\dot{x}}^{\prime }=\left[\begin{array}{c} \dot{x}\\ \dot{\xi } \end{array}\right]=\left[\begin{array}{ccc} A-\mathrm{BK} & \; & Bk_{I}\\ DK-C & \; & -Dk_{I} \end{array}\right]\left[\begin{array}{c} x\\ \xi \end{array}\right]+\left[\begin{array}{c} 0\\ 1 \end{array}\right]r$$

where $x^{\prime }$ is the augmented state vector, and ${\dot{x}}^{\prime }$ is its derivative:

(20) $$x^{\prime }={\left[\begin{array}{ccccc} \phi & \delta & \dot{\phi } & \dot{\delta } & \xi \end{array}\right]}^{⊤}$$

We can now define $K^{\prime }=\left[\begin{array}{cc} K & -k_{I} \end{array}\right]$. Therefore, we can rewrite Equation (17) as follows:

(21) $$u=-K^{\prime }x^{\prime }$$

The values of Q and R used in this work for Equation (16) are:

(22) $$Q={\left[\begin{array}{ccccc} 0 & 0 & 1\times 10^{-1} & 0 & 500 \end{array}\right]}^{⊤}\;\;,\;\;R=1\times 10^{-4}$$

These values were adjusted by trial and error to obtain a realistic behavior.

The $K^{\prime }$ values obtained for a 20 m/s forward velocity can be seen in Equation (23). It was not necessary to calculate $K^{\prime }$ values for different velocities, as could be expected, since the controller works fine with the values obtained for the forementioned velocity for all the speed range used in this work.

(23) $$K^{\prime }=1\times 10^{3}\;\left[\begin{array}{ccccc} -1.1768 & 0.1063 & -0.2079 & 0.0189 & 2.2361 \end{array}\right]$$

Now, the controller target, which represents the control input (*u*), should be assigned. Hence, some terms are calculated, such as position ($u_{i}$), velocity ($v_{i}$), and angular errors ($\alpha_{i}$) between the motorcycle position and the spline curve:

(24) $$\begin{array}{ccc} u_{i} & = & \cos\psi \;\left(y_{sp}-y_{rp}\right)-\sin\psi \;\left(x_{sp}-x_{rp}\right) \end{array}$$

(25) $$\begin{array}{ccc} v_{i} & = & {\mathrm{sp}}_{n}\times {\mathrm{rp}}_{vel} \end{array}$$

(26) $$\begin{array}{ccc} \alpha_{i} & = & \alpha -\psi \end{array}$$

where ψ stands for the yaw motorcycle angle, $x_{sp}$ and $y_{sp}$ are the spline coordinates, $x_{sp}$ and $y_{sp}$ are the reference point coordinates mentioned in Equations (2) and (3), ${\mathrm{sp}}_{n}$ is the spline normal vector, ${\mathrm{rp}}_{vel}$ is the reference point velocity, and α is the angle of the spline tangent vector. With these values,

(27) $$\begin{array}{c} K_{1}=-k_{1}\;u_{i} \end{array}$$

(28) $$\begin{array}{c} K_{2}=-k_{2}\;v_{i} \end{array}$$

(29) $$\begin{array}{c} K_{3}=-k_{3}\;\alpha_{i} \end{array}$$

Finally, the roll target expression is obtained:

(30) $$\phi_{target}=K_{1}+K_{2}+K_{3}$$

The steer torque expression becomes:

(31) $$\tau_{steer}=-K^{\prime }\;x^{\prime }+k_{d}\;v_{steer}$$

The second term in Equation (31) acts as a steer damper, in order to minimize all the small instabilities coming from different sources (contact forces, tire forces, controller, etc.), achieving a better performance without becoming slow on response. The variable $v_{steer}$ refers to the steer velocity, while $k_{d}$ is a damping coefficient, which, in our case, takes a value of 10 Ns/rad.

#### 2.3.4. Torso Controller

**Figure 4:**
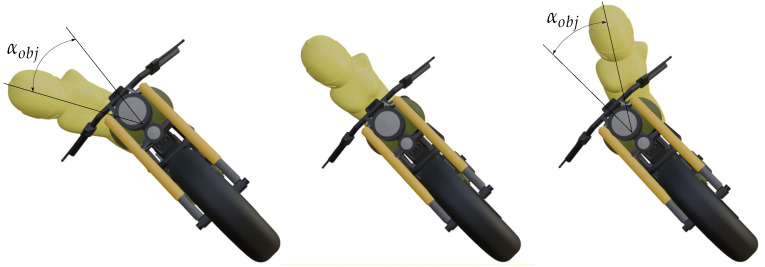
Inside, neutral, and outside configurations in a torso controller.

Torso roll movement has a capital influence on the motorcycle dynamics, since it changes the center of mass of the vehicle. If the torso goes inside a turn, the motorcycle lean angle can be reduced, whereas going outside instead forces the motorcycle to increase its roll angle to tackle the same turn at the same speed. In order to analyze this effect in the estimator, a torso controller was implemented. The controller allows to configure three different positions as seen in Figure 4: inside position, neutral position, and outside position.

In the neutral position, the torso has no influence in the maneuver, since it stays aligned with the motorcycle. On the other hand, the inside and outside positions modify the relative angle between the torso and the motorcycle, changing the center of mass position. The input angle for the controller is defined by Equation (32).

(32) $$\alpha_{obj}=0.5\;F\;\phi$$

where ϕ is the vehicle roll angle, *F* is a factor that can take three values: 0 for a neutral position, 1 for an inside position, and −1 for an outside position. The 0.5 was used in this work, but other values could be used if more or less rider lean is desired. Once obtained, a PD controller can be defined as:

(33) $$\begin{array}{ccc} \tau_{torso} & = & K\;\epsilon -C\;{\dot{\alpha }}_{torso} \end{array}$$

(34) $$\begin{array}{ccc} \epsilon & = & \alpha_{obj}-\alpha_{torso} \end{array}$$

Proportional (*K*) and derivative (*C*) term values used in this work are 1.5 × 10${}^{3}$ and 1 × 10${}^{3}$.

### 2.4. Sensor Models

As described in [11], it is assumed that wheel speed sensor and angular rate measurements are available. These sensors are already available in many commercial motorcycles. Sensor data is built from the values of the multibody simulation.

In order to obtain vehicle forward speed, a wheel speed sensor is employed. Through it, longitudinal speed can be easily estimated by Equation (35). Values from any wheel can be taken, since there is no significant difference between them, except during aggressive accelerating or braking phases. When the wheel slides, the speed estimation will not be correct. Moreover, when the vehicle is tilted, the effective radius of the wheel is reduced. The multibody simulation used in this work can represent these two situations.

(35) $$v_{long}=\omega_{wheel}\;r_{wheel}$$

**Figure 5:**
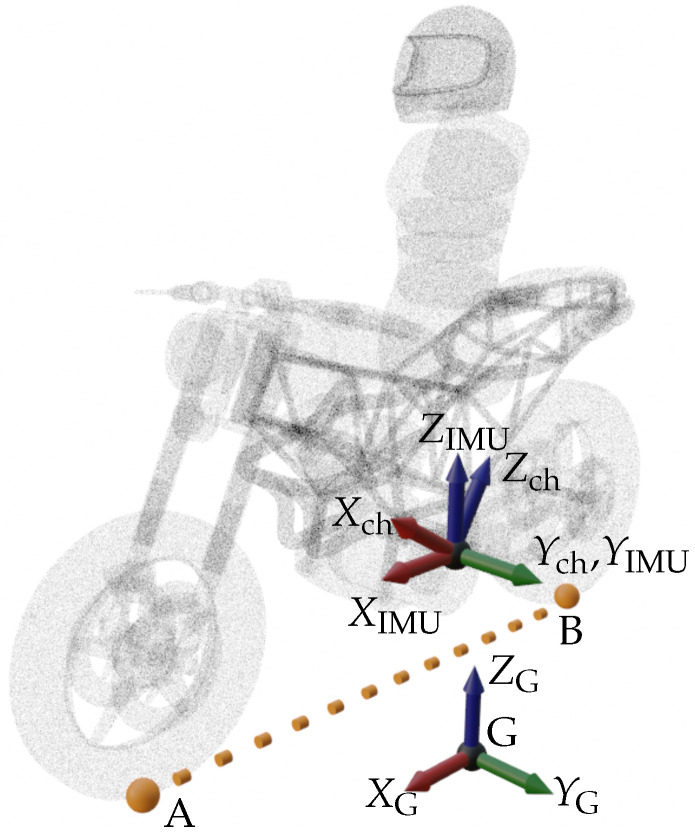
IMU position adjust.

An Inertial Measurement Unit (IMU) sensor was modeled in order to obtain angular rate measurements from the chassis element. The sensor is attached to the chassis reference frame, but in order to obtain the correct measurements, the IMU longitudinal axis should be aligned with the roll axis of the vehicle. This is particularly important since if this condition is not fulfilled, angular rate values will not be the correct ones. A rotation matrix can be applied to the sensor system reference in order to fix the possible wrong alignment between the IMU axis and the longitudinal axis of the motorcycle. This can be seen in Figure 5. Through Equation (36), the right angular rate values can be obtained.

(36) $$\omega_{\mathrm{IMU}}=R_{\mathrm{IMU}}^{⊤}\;R_{\mathrm{ch}}^{⊤}\;\omega_{\mathrm{ch}}$$

where $R_{\mathrm{IMU}}$ is the rotation matrix of the IMU relative to the chassis reference system, and $R_{\mathrm{ch}}$ is the rotation matrix of the chassis reference system with respect to the global axes. Even if the IMU is perfectly aligned when the motorcycle goes at constant speed in straight line, there will be some situations in which the axis coincidence condition will not be fulfilled.

For example, during a turn, when the motorcycle leans, the wheel contact points change their position due to the width difference between the two tires (in this case, as usual, the rear tire is wider than the front one). When the steering is turned, the front wheel contact point changes due to the fork trail, which also leads to a misalignment. Suspension movements can also produce similar effects. In all of these situations, an error between the IMU longitudinal axis and the motorcycle roll axis is expected, but it is an unavoidable fact.

In the case of both sensors, in order to achieve a more realistic situation, some additive random noise is added to the data in order to mimic real sensor characteristics. The noise is modeled as additive Gaussian white noise. However, neither the plant nor the measurement white noise. Therefore, for both sensors, values employed in the estimator (obs) are obtained through the simulation ones (mb) as:

(37) $$\begin{array}{ccc} \omega_{\mathrm{IMU}}^{\mathrm{obs}} & = & \omega_{\mathrm{IMU}}^{\mathrm{mb}}+N_{\mathrm{IMU}} \end{array}$$

(38) $$\begin{array}{ccc} \omega_{\mathrm{ws}}^{\mathrm{obs}} & = & \omega_{\mathrm{ws}}^{\mathrm{mb}}+N_{\mathrm{ws}} \end{array}$$

where $N_{\mathrm{IMU}}$ and $N_{\mathrm{ws}}$ are pseudorandom numbers following a normal distribution with 0 mean, which simulate the noise of the IMU and the wheel speed sensor, respectively. In the case of the IMU, the standard deviation used for the simulated noise is 9.839 × 10${}^{-4}$ rad/s (this value was obtained from a measurement taken with a low-cost IMU). For the wheel speed sensor, since no data was available, a conservative estimation of 1 m/s was taken for the standard deviation of the sensor noise.

### 2.5. Scenarios and Maneuvers

**Figure 6:**
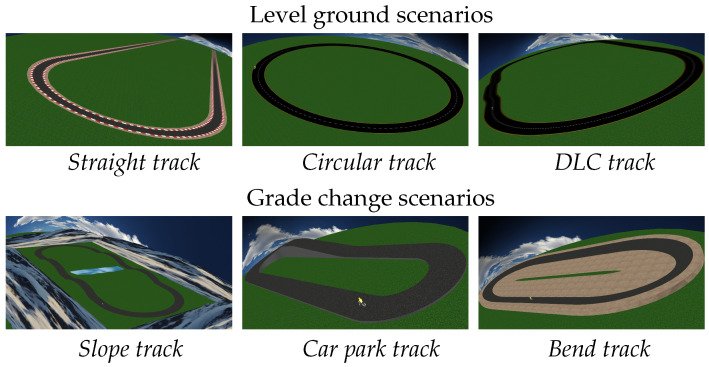
Scenarios created to test the estimator behavior.

The results from the previous work shown in [11] were obtained for a bicycle with no suspensions. Therefore, the results were only verified for low speed, thin tires, and without suspensions. Moreover, due to the experimental setup, the motion of the rider was unknown, and the measurement of the roll angle for verification purposes could only be performed on flat surfaces. Since this work is based on a simulation, both the scenario slope and bank angle can be controlled, and every magnitude can be measured, including the motion of the rider, which is part of the model. Some scenarios were built in order to test the estimations. The goal is to check the behavior of the estimator in all kind of situations, such as level grounds, slopes or bends. With this in mind, six scenarios are proposed, as shown in Figure 6: three with level ground and another three with slopes and/or bank angles.

The level ground scenarios are the *Straight track*, *Circular track*, and *DLC track* scenarios, while the *Slope track*, the *Car park track*, and the *Bend track* are non-planar tracks.

The *Straight track* is actually an oval track with two 2 km long straights, intended to study high-speed maneuvers and the transition from straight to turn. The *Circular track* is almost circular (it was approximated by four splines, and thus it is not a perfect circumference) to verify the steady-state cornering behavior of the observer. In this case, it has a radius of 50 m. The *DLC track* is also an oval track, but it features a double lane change in one of its straights.

When considering the non-planar tracks, all of them are ovals. The *Slope track* has two large bumps on each straight. Each one reaches a height of 6.5 m over a length of 38.4 m, which gives an average slope of 17%. After they reach their maximum height, they go back to the base level. The *Car park track* has an ascending slope combined with a turn, with a trajectory resembling a screw thread. The slope of this part is 22%. After that, there is a straight descent with a slope of 18% until the initial level is achieved. Finally, the *Bend track* is a flat circuit, but with a bank angle of 15${}^{\circ }$. The range of speeds used for the maneuvers reproduced in every scenario are shown in Table 3. The full data set obtained from the simulations is provided as supplementary material with this paper.

## 3. Roll Angle Estimator

The tool described in this section aims at estimating the roll angle of a STV with respect to gravity. To achieve that, it is assumed that the data from previously described sensors are available. Those sensors are affordable and easy to install on a vehicle, but they do not provide any direct measurement of the roll angle. To resolve this issue, information from both sensors and knowledge from the system has to be blended. In this work, a Kalman filter algorithm is employed to develop two variants of the roll angle estimator.

### 3.1. The Kalman Filter

The Kalman filter is a stochastic estimator that combines predictions from a model with measurements coming from sensors. The equations of the discrete version of Kalman filter are reproduced here. The reader interested in a deeper understanding of the Kalman filter is referred to any of the books on the topic, such as [22,23].

The filter runs in two stages: prediction and correction. During the prediction phase, the state x and the covariance matrix of its estimation error P are propagated by means of the model:

(39) $$\begin{array}{ccc} {\hat{x}}_{k}^{-} & = & F{\hat{x}}_{k-1}^{+}+Gu_{k-1} \end{array}$$

(40) $$\begin{array}{ccc} P_{k}^{-} & = & F_{k-1}P_{k-1}^{+}F_{k-1}^{⊤}+\Sigma^{P} \end{array}$$

where F stands for the transition model of the system, G is the input matrix, ${\hat{x}}_{k}^{-}$ is the estimation of the state vector in time step *k* before the measurement is applied, ${\hat{x}}_{k-1}^{+}$ is the estimation of the state vector of the k−1 time step after the corresponding measurements were applied, and $u{}_{k}$ is the input of the system. If measurements from sensors are available, they are used at the correction phase to improve the estimation from the prediction stage.

First, innovation $\tilde{y}$ is calculated as the difference between the measurements from sensors ($o_{k}$) and the expected sensor readings according to the model ($H{\hat{x}}_{k}^{-}$). Kalman gain (K) and the innovation covariance matrix (S):

(41) $$\begin{array}{ccc} {\tilde{y}}_{k} & = & o_{k}-H{\hat{x}}_{k}^{-} \end{array}$$

(42) $$\begin{array}{ccc} S_{k} & = & HP_{k}^{-}H^{⊤}+\Sigma^{S} \end{array}$$

(43) $$\begin{array}{ccc} K_{k} & = & P_{k}^{-}H^{⊤}S_{k}^{-1} \end{array}$$

The value of S represents the uncertainty in the system state projected via the sensor function ($HP_{K}^{-}H^{⊤}$) plus an additional uncertainty, $\Sigma^{S}$, due to the sensor noise. Finally, the estimation of the state and its covariance are updated by means of the Kalman gain:

(44) $$\begin{array}{ccc} x_{k}^{+} & = & x_{k}^{-}+K_{k}{\tilde{y}}_{k} \end{array}$$

(45) $$\begin{array}{ccc} P_{k}^{+} & = & \left(I_{g}-K_{k}H\right)P_{k}^{-} \end{array}$$

If measurements from sensors are not available, the correction stage is omitted.

### 3.2. Dynamical Model of the Filter

The model employed by the filter has two states: the roll angle and the bias of the angular rate sensor along the body fixed *x*-axis. The relationship among the angular rates measured by the body-mounted angular rate sensors ($\omega_{b}^{B}$, $\omega_{y}^{B}$, $\omega_{z}^{B}$) and the time derivative of the roll ($\dot{\phi }$), pitch ($\dot{\theta }$), and yaw ($\dot{\psi }$) angles of the vehicle body follows from:

(46) $$R^{⊤}\dot{R}={\tilde{\omega }}^{B}=\left[\begin{array}{ccc} 0 & -\omega_{z}^{B} & \omega_{y}^{B}\\ \omega_{z}^{B} & 0 & -\omega_{x}^{B}\\ -\omega_{y}^{B} & \omega_{x}^{B} & 0 \end{array}\right]$$

and can be expressed as:

(47) $$\begin{array}{ccc} \dot{\phi } & = & \left(\omega_{y}^{B}\sin\phi +\omega_{z}^{B}\cos\phi \right)\;\tan\theta +\omega_{x}^{B} \end{array}$$

(48) $$\begin{array}{ccc} \dot{\theta } & = & \omega_{y}^{B}\cos\phi -\omega_{z}^{B}\sin\phi \end{array}$$

(49) $$\begin{array}{ccc} \dot{\psi } & = & \frac{\omega_{y}^{B}\sin\phi +\omega_{z}^{B}\cos\phi }{\cos\theta } \end{array}$$

Assuming a small pitch angle, |θ|≈0, Equation (47) becomes:

(50) $$\dot{\phi }\approx \omega_{x}^{B}$$

The bias ($b_{x}$) of the *x* angular rate sensor ($\omega_{x}^{B}$) can be modeled as a random walk, i.e., assuming that it is constant and that the variations are produced by the plant noise. Once $b_{x}$ is known, $\omega_{x}^{B}$ can be corrected. Therefore, after applying the forward Euler integration method, the dynamic model of the filter becomes:

(51) $${\left[\begin{array}{c} \hat{\phi }\\ {\hat{b}}_{x} \end{array}\right]}_{k}^{-}=\left[\begin{array}{cc} 1 & -dt\\ 0 & 1 \end{array}\right]{\left[\begin{array}{c} \hat{\phi }\\ {\hat{b}}_{x} \end{array}\right]}_{k-1}^{+}+\left[\begin{array}{c} dt\\ 0 \end{array}\right]\omega_{x,k-1}^{B}$$

where the states at the present time step *k* are expressed as a function of the states and the inputs of the previous time step k−1, being dt the integration time step. In the previous equation we can identify the system and input matrices (F and G) as follows:

(52) $$F=\left[\begin{array}{cc} 1 & -dt\\ 0 & 1 \end{array}\right]$$

(53) $$G=\left[\begin{array}{c} dt\\ 0 \end{array}\right]$$

### 3.3. Absolute Measurements of the Roll Angle

In the correction stage of this Kalman filter, absolute measurements of the roll angle are needed. None of the sensors considered in this work provides a roll angle measurement, therefore, a model was built from the sensor measurements, and employed as absolute roll angle measurement. This is the measurement employed at the Kalman filter correction stage, denoted as $o_{k}$ in Equation (41). The model is the same as described in [11], and is created by combining two ways of obtaining a roll angle estimation, coming from two different assumptions. The first one is obtaining from the steady-state cornering equilibrium, which will be referenced as $\phi_{d}$ on this work:

(54) $$\phi_{d}=\arctan\left(\frac{\dot{\psi }v}{g}\right)$$

where *v* is the forward vehicle speed (obtaining from wheel speed sensor), *g* is the gravity acceleration, and $\dot{\psi }$ is the yaw velocity, which is assumed that $\omega_{z}^{B}$ (obtaining from IMU) is an enough accurate estimation. This method works well for small roll angles on level roads, but it tends to underestimate the roll angle in more realistic conditions due to the gyroscopic effect and the thickness of the tires, which are not considered in this model.

On the other hand, roll angle can be obtained under a null pitch rate condition, which means the Equation (48) becomes:

(55) $$0=\omega_{y}^{B}\cos\left(\phi \right)-\omega_{z}^{B}\sin\left(\phi \right)$$

and then, the another estimation of the roll angle, referenced as $\phi_{\omega }$ in this case, can be calculated as:

(56) $$\phi_{\omega }=\arctan\left(\frac{\omega_{y}^{B}}{\omega_{z}^{B}}\right)=sgn\left(\omega_{z}^{B}\right)\arcsin\left(\frac{\omega_{y}^{B}}{\sqrt{{\left(\omega_{y}^{B}\right)}^{2}+{\left(\omega_{z}^{B}\right)}^{2}}}\right)$$

where $sgn(\omega_{z}^{B})$ is the sign of the *z* angular rate. This method is more convenient for greater roll angles because the predicted roll tends to oscillate around the true value, although is noisier than the steady-state based one. In order to take the best from both estimations, they are combined using a weighted mean. The weighing function changes its value depending on the last available estimation provided by the equations of the steady-state cornering equilibrium, $\phi_{d}$.

**Figure 7:**
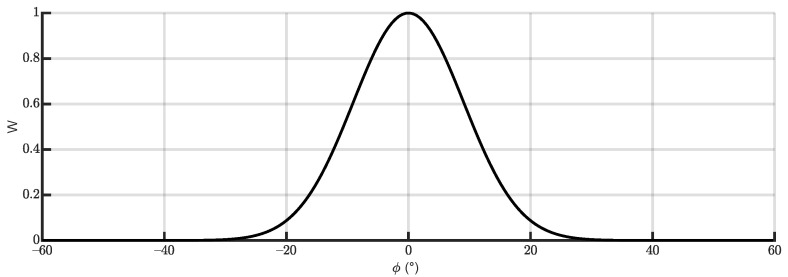
Shape of the weight function used to combine the two estimated measurements of the roll angle.

This is the main difference with respect to the method employed in [11], where the last estimation provided by the Kalman filter ($\hat{\phi }$) was used instead. The change was motivated because it was seen that, in some cases, an instantaneous wrong angle estimation could lead to a inadequate weighting value, which could eventually make the estimation diverge definitively from the true value. In this work, the weighting function, which can be seen in Figure 7, is defined by:

(57) $$W=\exp\left(\frac{-\phi_{d}^{2}}{{\bar{\phi }}^{2}}\right)$$

where ${\bar{\phi }}^{2}$ is a constant value that can be used to adjust the behavior of the weighting function. In this work, a value of ${\bar{\phi }}^{2}=0.04$ was used, with the dynamically estimated roll angle $\phi_{d}^{2}$ expressed in radians.

Therefore, the roll angle measurement $o_{k}$ to be employed in Equation (41) is $\phi_{m}$, built as weighted combination of $\phi_{d}$ and $\phi_{\omega }$:

(58) $$\phi_{m}=W\phi_{d}+\left(1-W\right)\;\phi_{\omega }$$

Since this measurement is the same magnitude than the first component of the state vector, the output matrix H results as follows:

(59) $$H=\left[\begin{array}{cc} 1 & 0 \end{array}\right]$$

### 3.4. Adaptation of the Kalman Filter for Colored Noise

The Kalman filter was formulated assuming that the noise affecting both the plant and the measurements is additive white Gaussian noise. However, neither the plant nor the measurement noises are white for the models used in this work. Instead, the lower frequencies are predominant in both noises (for instance, if the measurement is overestimating the roll angle at a given moment, it is more probable than the next time step it will be also overestimating it). This kind of behavior, usually known as colored noise, can be modeled as follows [22]:

(60) $$Cn_{i+1}=W\times Cn_{i}+Wn_{i}$$

where the subindex *i* represents the time step, Cn is the colored noise, Wn is the white noise, and *W* is a parameter that can take any value from 0 to 1 to modulate the influence of the previous value of the noise. If *W* = 0, the model would produce white noise. If *W* = 1, the noise would behave as a Markov model. If we introduce this noise model for the noise of the roll angle of the dynamic model of the Kalman filter, and also for the noise of the sensor, the resulting filter matrices will be as follows:

(61) $$F^{\prime }=\left[\begin{array}{cccc} 1 & -dt & 0 & 1\\ 0 & 1 & 0 & 0\\ 0 & 0 & W_{1} & 0\\ 0 & 0 & 0 & W_{2} \end{array}\right]$$

(62) $$G^{\prime }=\left[\begin{array}{c} dt\\ 0\\ 0\\ 0 \end{array}\right]$$

(63) $$H^{\prime }=\left[\begin{array}{cccc} 1 & 0 & 1 & 0 \end{array}\right]$$

where $F^{\prime }$, $G^{\prime }$, and $H^{\prime }$ are the system, input, and output matrices of this augmented filter, respectively, $W_{1}$ is the weighting parameter of the measurement noise, and $W_{2}$ the weighting parameter of the plant noise (applied only to the first state of the filter). When this model is applied, the measurement noise is incorporated into the colored noise, and, therefore, the covariance matrix of the measurement noise should be set to zero. Other than that, the method is identical to the previous one.

## 4. Results and Discussion

One of the key aspects of the performance of a Kalman filter is a proper adjustment of the values of the plant and the measurement noise covariance matrices. In the algorithm presented in this work, the weighting parameter for the measurement estimation also has to be set. Moreover, in the filter adapted to deal with colored noise there are two additional parameters, i.e., the weights used to model low frequency component of the noise. In this work, this tuning process was made by trial and error, seeking the minimization of the Root Mean Square Estimation Error (RMSEE) of the worst maneuver. The parameters used in this work are shown in Table 4.

**Figure 8:**
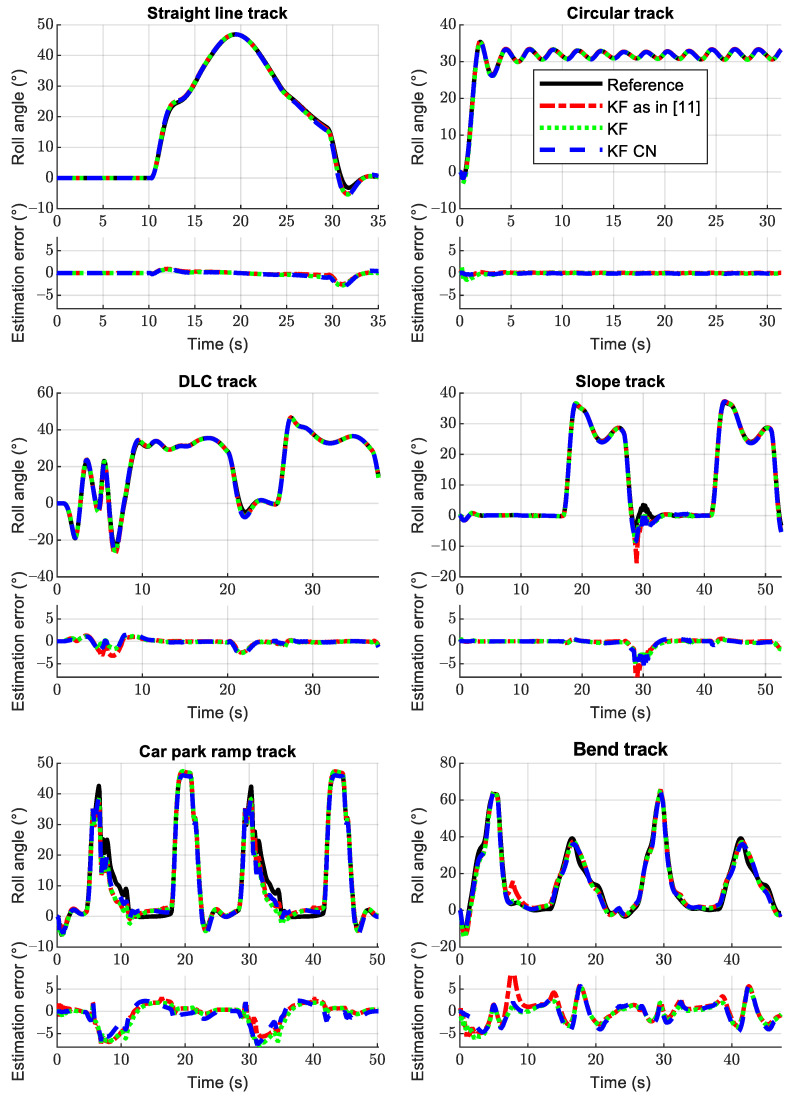
Estimated roll angle and roll angle error for the six different scenarios. All of these tests were performed with the rider in a neutral position. The reference is the black solid line, the red dash dotted line is the Kalman filter as presented in [11], the green dotted line is the variation of that Kalman filter presented here, and the blue dashed line represents the Kalman filter adapted for colored noise.

The results obtained for all the six tests scenarios are shown in Figure 8. The numeric results of the RMSEE for both methods are shown in Table 5. The method as described in [11] is also added to the comparison. In all these tests, the rider kept a neutral position. The results are very good for all the maneuvers with flat floor, with a RMSEE below 1${}^{\circ }$ for all estimation methods. The maneuvers on inclined floors are a harder challenge for the estimator, mainly the *Car park ramp track* and the *Bend track*. In these maneuvers the RMSEE are around 2.5${}^{\circ }$, with the Kalman filter for colored noise having slightly better estimations for both maneuvers.

With different parameters, the results of both maneuvers can be improved, but improving the results in one of the maneuvers would produce deterioration of the performance on the other. The estimation at the *Car park ramp track* is, in general, good, but the accuracy degrades after the turns. This might be because the bumps of this track start after the turns and the motorcycle goes over them when it is still leaned, thus degrading the performance of all the indicators, because neither the pitch nor the pitch rate are null.

The estimation method as presented in [11] provides good estimations in general, but it seems to be less robust than the new variations, as can be seen in the plots for the maneuvers in the *Slope track* and *Bend track*, where some short but important deviations can be appreciated. The risk with this method is that a temporal wrong estimation can lead to an inadequate weighting value to combine the two roll angle estimations used at the correction phase of the filter. This can potentially turn into a positive feedback that could make the estimation diverge from the true value.

The Kalman filter adapted for colored noise produces the best worst-case scenario estimations, but only by a narrow margin, and it is not the best in all the maneuvers. Moreover, it is more difficult to adjust because it has more parameters to tune, and thus it does not have any practical advantage in its current form.

Nevertheless, there is a potential benefit from using the colored noise adaptation for the filter, although it is not explored in this work. Since the characterization of the noise is improved, the statistical characteristics of the innovation sequence would be improved as well, thus allowing to use innovation-based adaptive Kalman filters, which should be able to improve the performance of the method for all the maneuvers that have now the worst estimation results. It was recently shown that innovation-based Kalman filters can work reasonably well even if the statistical properties of the noise are not perfect, and even with nonlinear models [24].

**Figure 9:**
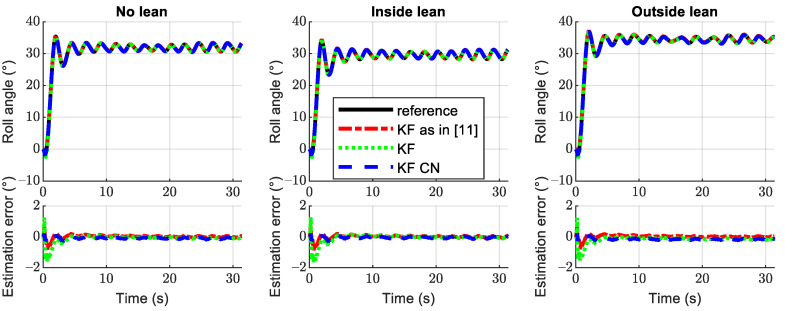
Estimated roll angle and roll angle error for the *Circular test track*. The left plot represents the maneuver with the rider in a neutral position, the central plot represents the rider tilting inwards during the turn, and the right plot represents the rider tilting outwards during the turn.

Another point that we wanted to address with this work was the influence of the rider position on the roll angle estimation error. For the sake of conciseness, only the *Circular track* was studied. Therefore, three simulations were run on this track, the only difference being the driver keeping its neutral position, leaning inwards, or leaning outwards. The results are shown in Figure 9, and the numerical results of the RMSEE are shown in Table 6. As expected, when the rider tilts inwards, the roll angle to perform a given maneuver is reduced, and the opposite happens when the rider leans outwards. Regarding the roll angle estimation accuracy, the difference between the different rider positions does not produce any significant perturbation.

## 5. Conclusions

This paper is based on the roll angle estimation algorithm presented in [11], where it was first presented and tested at low speed with an instrumented bicycle. However, due to technical limitations of the experimental setup, part of the validation could not be performed. This work aims at verifying the performance of the roll angle observer in more challenging conditions. In order to achieve this goal, a multibody model of a motorcycle was developed, including toroidal-shaped wheels to consider the displacement of the contact point with wide tires. The multibody model also considers the suspensions, whose movement produces misalignments that could potentially degrade the performance of the roll angle observer. In addition, non planar scenarios modeled as triangle meshes, and rider motion were also considered. Finally, higher speeds than those considered in [11] were tested here.

The multibody model, governed by a longitudinal controller in charge of keeping an adequate speed and by a lateral controller used to keep the balance of the motorcycle and follow the prescribed trajectories, performed maneuvers in six different scenarios. From these maneuvers, some measurements were obtained, mimicking the properties of actual sensors by adding some white Gaussian noise. These measurements were used to verify the performance of the state observer, which was slightly modified to improve the robustness.

Moreover, an augmented version of the observer was devised to deal with the colored noise present at the plant and measurements used at the Kalman filter. Although this last observer provided slightly better results than the simpler version assuming white noise, the improvement is so subtle that in its current state, it would not be justified its practical use, given the more difficult tuning process and the increase in the size of the problem.

However, this new observer should provide (with the proper parameter tuning) statistical properties more consistent with the theoretical assumptions made to develop the Kalman filter. Therefore, the innovation sequence of the Kalman filter could be used to implement an innovation-based adaptive Kalman filter, which, in turn, should provide better estimation results, specially for the maneuvers that have the worst results in the present paper.

## Figures and Tables

**Table 1:** Mass and inertial properties of all the model elements.

Element	Mass (kg)	Inertia Tensor [Ix Iy Iz Ixy Ixz Iyz] (kg m²)					
Chassis	165.13	6.14	12.51	8.55	0.01	−2.43	0.03
Swingarm	8	0.10	0.23	0.31	0	−0.01	0
Rear wheel	14.7	0.43	0.81	0.43	0	0	0
Front wheel	11.9	0.32	0.62	0.32	0	0	0
Steer & Fork bottles	10.21	0.23	0.11	0.16	0	−0.01	0
Fork bars	3.13	0.05	0.02	0.03	0	0	0
Torso	42	2.32	2.13	0.49	0	−0.23	0

**Table 2:** Whipple’s model parameters employed.

Parameter	Symbol	Value, Unit
wheelbase	*w*	1.3295 m
trail	*c*	0.0696 m
steer axis tilt	λ	0.4363 rad
gravity	*g*	9.81 m/s²
forward velocity	*v*	20 m/s
rear wheel		
radio	$r_{R}$	0.3069 m
mass	$m_{R}$	14.7 kg
mass moments of inertia	($I_{Rxx}$, $I_{Ryy}$)	(0.4332, 0.8134) kg m²
front wheel		
radio	$r_{F}$	0.2819 m
mass	$m_{F}$	11.9 kg
mass moments of inertia	($I_{Fxx}$, $I_{Fyy}$)	(0.3265, 0.6209) kg m²
rear body, chassis and torso		
position left of mass	($x_{B}$, $z_{B}$)	(0.6344, −0.4741) m
mass	$m_{B}$	215.13 kg
mass moments of inertia	$\left[\begin{array}{ccc} I_{Bxx} & 0 & I_{Bxz}\\ 0 & I_{Byy} & 0\\ I_{Bxz} & 0 & I_{Bzz} \end{array}\right]$	$\left[\begin{array}{ccc} 22.3589 & 0.0197 & -2.0664\\ 0.0197 & 29.6528 & 0.0316\\ -2.0664 & 0.0316 & 10.3619 \end{array}\right]$ kg m²
front handlebar and fork assembly		
position left of mass	($x_{H}$, $z_{H}$)	(1.0669, −0.7446) m
mass	$m_{H}$	13.3516 kg
mass moments of inertia	$\left[\begin{array}{ccc} I_{Hxx} & 0 & I_{Hxz}\\ 0 & I_{Hyy} & 0\\ I_{Hxz} & 0 & I_{Hzz} \end{array}\right]$	$\left[\begin{array}{ccc} 0.4221 & -0.0002 & -0.1112\\ -0.0002 & 0.3457 & 0.0002\\ -0.1112 & 0.0002 & 0.2722 \end{array}\right]$ kg m²

**Table 3:** Speeds used for the maneuvers in every test track (in m/s).

Track	Minimum Speed	Average Speed	Maximum Speed
Straight	29.00	38.15	50.00
Circular	15.00	15.88	16.21
DLC	10.00	19.38	23.00
Slope	5.79	12.62	16.41
Car park	3.84	8.41	15.00
Bend	8.78	11.58	15.00

**Table 4:** Values of the parameters used in this work (in SI units). In this work, both the simple Kalman filter presented here, and the previous version in [11] were used with the same parameters for a fair comparison. Note that the $\Sigma^{S}$ for the Kalman filter for colored noise is null. The reason is that the measurement noise is contained in the third row of the plant noise.

Parameter	$\Sigma^{P}$	$\Sigma^{S}$	${\bar{\phi }}^{2}$	Noise Weights
**Kalman filter**	$\left[\begin{array}{cc} 5\times 10^{-7} & 0\\ 0 & 1\times 10^{-8} \end{array}\right]$	1.5	0.04	-
**Kalman filter for colored noise**	$\left[\begin{array}{cccc} 1\times 10^{-6} & 0 & 0 & 0\\ 0 & 1\times 10^{-8} & 0 & 0\\ 0 & 0 & 0.5 & 0\\ 0 & 0 & 0 & 1\times 10^{-6} \end{array}\right]$	0	0.04	$W_{1}=0.8$ $W_{2}=0.5$

**Table 5:** Root mean square estimation errors for the three estimation algorithms on the six scenarios (in degrees).

Maneuver	Straight	Circular	DLC	Slope	Car Park	Bend
**Kalman filter as in [11]**	0.60	0.12	0.91	1.21	2.50	2.71
**Kalman filter**	0.69	0.27	0.71	0.90	2.81	2.28
**Kalman filter for colored noise**	0.78	0.11	0.82	1.05	2.34	2.35

**Table 6:** Root mean square estimation errors for the three estimation algorithms on the *Circular track* with the rider in neutral position, leaning inwards, and leaning outwards (in degrees).

Maneuver	Neutral	Inwards	Outwards
**Kalman filter as in [11]**	0.12	0.13	0.12
**Kalman filter**	0.27	0.28	0.28
**Kalman filter for colored noise**	0.11	0.10	0.17

## Data Availability

Data from the simulations are provided as supplementary material.

## Supplementary Materials

Data from the simulations are provided as supplementary material at https://www.mdpi.com/article/10.3390/s21196626/s1. The data are organized in .csv files, and a text file (readme.md) provides the description of the data.
